# Who, When, Why?—Traumatological Patients in the Emergency Department of a Maximum Care Provider

**DOI:** 10.3390/life13102046

**Published:** 2023-10-12

**Authors:** Jason-Alexander Hörauf, Cora Rebecca Schindler, Nils Mühlenfeld, Julian Zabel, Philipp Störmann, Ingo Marzi, Nicolas Söhling, René Danilo Verboket

**Affiliations:** Department of Trauma, Hand and Reconstructive Surgery, Goethe University Frankfurt am Main, Theodor-Stern-Kai 7, 60590 Frankfurt am Main, Germany; cora.schindler@kgu.de (C.R.S.); nils.muehlenfeld@kgu.de (N.M.); julian.zabel@kgu.de (J.Z.); philipp.stoermann@kgu.de (P.S.); ingo.marzi@kgu.de (I.M.); nicolas.soehling@kgu.de (N.S.); rene.verboket@kgu.de (R.D.V.)

**Keywords:** emergency department, trauma, crowding, urgent care

## Abstract

Nationwide, there is an annual increase in the number of patients in German emergency departments resulting in a growing workload for the entire emergency department staff. Several studies have investigated the situation in emergency departments, most of which were interdisciplinary, but there are no data on a solely traumatological patient population. The present study therefore aims to investigate the situation in a university-based trauma surgery emergency department. A total of 8582 traumatological patients attending a university hospital from 1 January 2019 to 31 December 2019 were studied. Various variables, such as reason for presentation, time of accident, diagnosis, and diagnostic as well as therapeutic measures performed were analyzed from the admission records created. The mean age was 36.2 years, 60.1% were male, 63.3% presented on their own to the emergency department, and 41.2% presented during regular working hours between 8:00 a.m. and 6:00 p.m., Monday through Friday. The most common reason for presentation was outdoor falls at 17.4%, and 63.3% presented to the emergency department within the first 12 h after the sustained trauma. The most common diagnosis was bruise (27.6%), and 14.2% of patients were admitted as inpatients. Many of the emergency room patients suffered no relevant trauma sequelae. In order to reduce the number of patients in emergency rooms in the future, existing institutions in the outpatient emergency sector must be further expanded and effectively advertised to the public. In this way, the emergency medical resources of clinics, including staff, can be relieved to provide the best possible care for actual emergency patients.

## 1. Introduction

Each year, more than 20 million patients present to emergency departments in German hospitals [1]. Over the recent decades, the annual patient volume in hospital emergency departments has steadily increased both in Germany and worldwide [2,3]. For German emergency departments, an annual increase in case numbers of up to 9% has been reported [4]. However, not all patients presenting to the emergency department require emergency care in the strict sense of the term. For example, O’Keeffe et al. reported that about one-third of patients who present to an emergency department did not require emergency care, and their health needs could also be met by common primary health care providers [5]. In addition to increasing health care costs, this leads to crowding in emergency departments, which in turn, can result in poorer treatment outcomes for the true emergency patient population due to a consecutive delay in diagnostic and therapeutic interventions [6]. Furthermore, overcrowded emergency departments also lead to a physical and psychological (over)load for the treating hospital staff, and patients waiting to be treated are unsatisfied with overcrowded waiting rooms and long wait times [7].

Traumatology is also seeing an increasing number of patients in emergency departments, even though the number of severely injured patients has remained constant in recent years [8]. Recently, Biberthaler et al. showed that 43% of all patients in the emergency department required treatment for musculoskeletal complaints [9]. These numbers emphasize the importance of traumatology as an essential component of emergency medicine.

Overall, however, data on traumatological patients presenting to German hospitals via the emergency department are limited. Therefore, the aim of the present retrospective study is to analyze the epidemiologic characteristics of traumatological patients presenting to the emergency department of a maximum care hospital. Based on the results and findings, the discussion will then focus on how alternative outpatient institutions can relieve the burden on emergency department staff.

## 2. Materials and Methods

### 2.1. Study Design and Data Acquisition

The present study is a retrospective study. Patients who presented independently or were admitted by ambulance to the traumatological emergency department of the University Hospital Frankfurt am Main between 1 January 2019 and 31 December 2019 were selected from the hospital’s internal documentation system (Orbis, Saarbrücken, Germany). In 2019, more than 9000 patients presented to the traumatological emergency department. For each patient, an admission record was created by the treating trauma surgeon and stored in the internal Orbis system. These admission records were analyzed in this study according to various characteristics, such as age and gender, reason for presentation, time of presentation, trauma sustained, referral, diagnostic and therapeutic measures performed (imaging, laboratory, wound care, surgery, immobilization of an extremity, inpatient admission, etc.), as well as the discharge diagnosis and main affected body region. All patients who present independently to the emergency department are screened and triaged by specially trained nursing staff between 05:00 a.m. and 21:00 p.m. to ensure that those patients with more severe injuries and/or injuries requiring acute care, such as lacerations, are presented promptly to a physician. Here, the patient’s pain situation is assessed using the Visual Analog Scale (VAS). To examine possible seasonal differences in the presentation of trauma patients to the emergency department, the months were grouped as follows: spring (March, April, May), summer (June, July, August), fall (September, October, November), and winter (December, January, February). During the aforementioned study period, 8582 traumatological patients were retrospectively analyzed.

The present study follows the STROBE (Strengthening The Reporting of Observational Studies in Epidemiology) guidelines for observational studies and the RECORD (Reporting of studies Conducted using Observational Routinely collected Data) guidelines for observational studies [10,11]. This study was approved by the local ethics committee (vote 19-491) of Johann Wolfgang Goethe University.

### 2.2. Statistical Analysis

The collected data were first assembled in Microsoft Excel version 16.63.1 (Redmond, WA, USA) and then imported into the software program Statistical Package for Social Sciences (SPSS) version 26.0 (SPSS Inc., Chicago, IL, USA), distributed by the software company International Business Machines Corporation (IBM, Armonk, NY, USA) for further descriptive and comparative statistical analyses. Frequencies are reported in both absolute numbers and percentages, rounded to one decimal place. Categorical variables were compared using the chi-square test. Continuous variables were compared using the Kruskal–Wallis test. A two-sided *p* value of <0.05 was assumed to be statistically significant. Metrically scaled data are expressed as the arithmetic mean ± standard deviation (SD).

## 3. Results

A total of 8582 traumatological patients treated in the emergency department were evaluated. Of the 8582 patients, 60.1% (n = 5159) were male and 39.9% (n = 3423) were female. The mean age was 36.2 years ± 22.1 years (standard deviation).

### 3.1. Time of Presentation

In all, 63.3% (n = 5429) of the patients presented on their own to the emergency department within the first 12 h after the trauma or were presented to the emergency department by the ambulance service, 9.7% (n = 833) presented to the ED at 12 to 24 h post trauma, another 7.1% (n = 605) presented at more than 24 h, 5.7% (n = 486) presented after more than 48 h, and 14.2% (n = 1229) presented after more than 72 h post trauma (total n = 8582) (Table 1).

The highest patient load occurred on Saturday (17.8%, n = 1529) and Sunday (15.9%, n = 1365), followed by Friday (14.1%, n = 1211), Wednesday (13.7%, n = 1173), Monday (13.5%, n = 1155), Tuesday (13.0%, n = 1117), and Thursday (12.0%, n = 1032), without statistical significance (*p* = 0.423) (Table 2). Approximately 41.2% (n = 3532) of all patients presented to the emergency department between 8:00 a.m. and 6:00 p.m., Monday through Friday, which is during regular working hours.

### 3.2. Reason for Presentation

The most common reason for presentation to the ED was outdoor falls in 17.4% of cases (n = 1495), followed by impact/bruising trauma in 15.3% (n = 1397), distortion trauma (of any region of the body) in 14.8% (n = 1270), and domestic falls in 10.0% (n = 854) (other reasons for presentation are shown in Table 3). Altogether, 12.3% (n = 1057) of patients suffered trauma while performing a sports activity. In 16.6% (n = 1425) of cases, the patients suffered trauma in the context of a work-related accident. Patients over 65 years of age fell significantly more often in the domestic environment (41.4% versus 5.5%, *p* < 0.001) than patients under 65 years of age. In turn, the latter suffered distortion trauma significantly more often than patients over 65 (16.4% versus 3.6%, *p* < 0.001).

### 3.3. Pain Level

Data on pain level quantified by VAS were available in 50.5% (n = 4337) of cases, with a mean VAS score of 3.1 points (±1.3 points SD). The minimum was a VAS score of 0 points, and the maximum was a VAS score of 8 points. Pain levels were most frequently reported with a VAS score of 2 points (32.0%, n = 1390) and 3 points (29.0%, n = 1256).

### 3.4. Admissions

In all, 20.2% (n = 1733) of the patients were taken to the ED by ambulance, another 5.8% (n = 504) were admitted via the trauma room, and 74.0% (n = 6345) presented to the ED on their own. Patients who were brought to the emergency department by the ambulance service were significantly more likely to have fractures than those who presented on their own (34.1% versus 15.8%, *p* < 0.001). In addition, patients who presented with the ambulance service were significantly more likely to undergo surgery (25.7% versus 7.3%, *p* < 0.001) and be admitted as inpatients (35.1% versus 7.1%, *p* < 0.001) than those patients who presented independently. A total of 8.4% (n = 725) of patients were referred to the ED by external medical colleagues [on-call service (so-called Ärztlicher Bereitschaftsdienst, ÄBD), private practitioners, or other hospitals]. Of these, 35.7% (n = 259) were referred by the ÄBD, 23.2% (n = 168) by a general practitioner, 30.5% (n = 221) by an external hospital, and 10.6% (n = 77) by an external resident specialist. Patients referred to the emergency department by external physicians (the planned/arranged transfers from external hospitals (n = 221) were excluded for the analysis) were not significantly more likely to undergo surgery (13.9% versus 9.8%, *p* = 0.109) and be admitted as inpatients (11.1% versus 12.7%, *p* = 0.589) than patients who came to the emergency department on their own or by ambulance. Patients older than 65 years were significantly more likely to be hospitalized than the younger comparison collective (39.4% versus 10.6%, *p* < 0.001).

### 3.5. Diagnostics

As part of the diagnostics performed in the ED, 16.6% (n = 1425) of the patients received a blood sample (for example, for the assessment of infection parameters such as C-reactive protein or leukocytes, or as a standard procedure preoperatively if the patient has undergone surgery immediately after admission). As standard radiological diagnostics, 57.3% (n = 4917) received conventional X-ray imaging. Additional computed tomography (CT) imaging (X-ray + CT) was required in 5.7% (n = 487) of cases, and additional magnetic resonance imaging (X-ray + MRI) was required in 0.5% (n = 42) of cases; 8.0% (n = 682) of patients received CT only, and 0.3% (n = 25) received MRI only. In 2.2.% (n = 185) of cases, imaging was already performed by the referring external physician. In 26.1% (n = 2244) of cases, according to the assessment of the treating physicians, no imaging was indicated.

### 3.6. Diagnoses and Affected Body Regions 

The most common diagnoses recorded in the ED were bruises in 27.6% (n = 2366) of cases, fractures in 19.2% (n = 1645) of cases, wounds in 16.6% (n = 1421) of cases, and distortion in 13.9% (n = 1194) of cases (other diagnoses are shown in Table 4).

**Figure 1:**
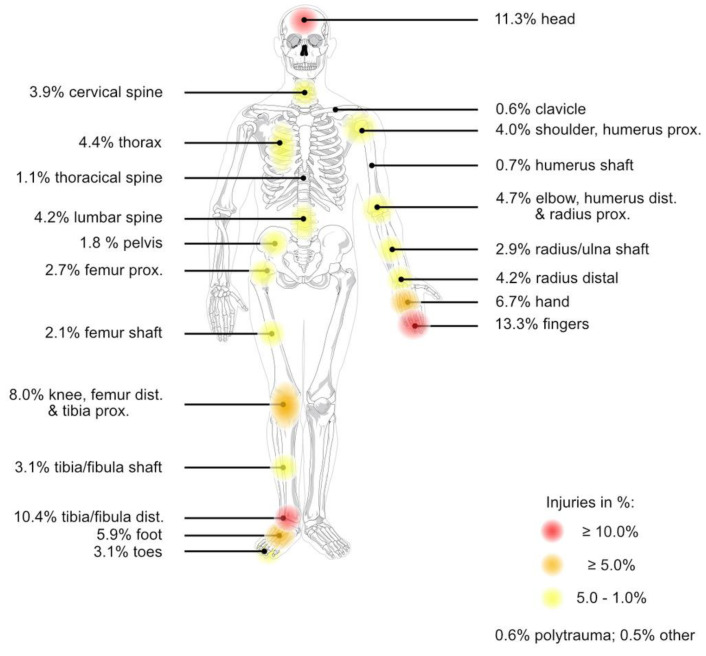
Shown are the frequencies of injuries to each affected body region (n = 8582; color-coded frequency categories: red ≥ 10.0%, orange ≥ 5.0%, yellow 5.0–1.0%). The most frequently affected body region was the fingers with 13.3% (n = 1138), followed by the head with 11.3% (n = 973), and the ankle or distal fibula/tibia with 10.4% (n = 896). The underlying schematic of a human skeleton was published by the artist Mariana Ruiz Villarreal under an unrestricted public license and made available in the public domain by the author.

The most commonly affected body region was the fingers in 13.3% (n = 1138) of cases, followed by the head with 11.3% (n = 973) and the ankle or distal fibula/tibia with 10.4% (n = 896) (the other affected body regions are shown in Figure 1).

### 3.7. Therapy

In 26.6% (n = 2282) of cases, surgical treatment of a wound (cleaning, suturing/gluing) or incision, for example of an abscess, was performed directly in the ED. In 35.6% (n = 3056) of cases, immobilization of an affected limb/joint was performed, for example, by application of a soft-cast splint, ankle orthosis, or Gilchrist bandage.

In 11.7% (n = 1004) of the cases, the injuries sustained required operative treatment. Of these, 48.8% (n =490) were treated as emergencies immediately (within 24 h) after diagnosis, 19.8% (n = 199) of the patients were hospitalized and underwent surgery in the further course of their stay, and 31.4% (n = 315) of the patients could be discharged after confirmation of the diagnosis and were then treated surgically later. Patients older than 65 years underwent surgery significantly more often than the younger comparison collective (27.0% versus 9.5%, *p* < 0.001).

### 3.8. Inpatient Admission

Overall, 14.2% (n = 1218) of patients presenting to the ED were admitted as inpatients for further therapy, and 85.8% (n = 7364) of patients could be discharged following treatment in the ED. The mean length of stay for inpatients was 7.5 days ± 7.4 days (SD).

### 3.9. Seasonality

During the study period, 22.3% (n = 1907) of patients presented in the spring, 28.6% (n = 2474) presented in the summer, 26.4% (n = 2258) presented in the fall, and 22.7% (n = 1943) of patients presented in the winter, with no significant difference in patient presentations between seasons (*p* = 0.392). The largest number of presentations (n = 1106, 12.9% of all cases) occurred in the month of June, and the smallest number (n = 486, 5.7% of all cases) in July, with this difference being significant (*p* = 0.031). The other comparisons between the number of presentations in each month did not reveal any significant differences. Regarding the reasons for presentation in the emergency department of a “fall outdoor” (*p* = 0.554), “fall indoor” (*p* = 0.965), and “distortion” (*p* = 0.850), there was no significant difference between seasons. Bicycle falls were significantly more frequent in summer than in winter (7.2% versus 2.2% of all reasons for presentation in each season, *p* = 0.020). Furthermore, there were no significant seasonal differences in the incidence of sports accidents (*p* = 0.541), emergency department admissions by ambulance service (*p* = 0.500), and incidence of fractures (*p* = 0.316).

## 4. Discussion

The present results demonstrate that the care of trauma surgery patients in the emergency department requires a large amount of human, spatial, and time resources. It is therefore all the more important not to overload the emergency department with an excessive number of patients.

One tool used to ease the burden on emergency departments is the expansion of outpatient emergency care. In recent years, for example, in addition to the existing specialists in private practice, the outpatient sector has been expanded to include institutions such as the medical on-call service ÄBD, which can be reached 24 h a day, 7 days a week, via a nationwide telephone number. The aim of these institutions is to provide adequate outpatient care for patients who do not require acute emergency care in a hospital or, if necessary, to refer them to a nearby hospital for further clarification. Unfortunately, more than 50% of the patients are not aware of these alternative outpatient emergency structures [3,12]. In a study by Somasundaram et al., only 11% of patients presenting to the ED had first contacted the ÄBD, who eventually referred them to the respective ED. At the same time, however, more than half of these patients would have visited an emergency outpatient facility if they had been aware of it [12]. An additional barrier to seeking outpatient care is the expectation of better care in the emergency department than in the physician’s office. A study by Northington et al. showed that even when a primary care provider in the sense of a general practitioner or resident specialist was known and available, nearly half of patients believed that medical care was better in an emergency department, even if it was associated with longer waiting times and higher personal financial costs [13].

Other frequently mentioned reasons for visiting the emergency department are closed offices and the lack of availability of (short-term) appointments in the private practice sector [14]. In our study, 41.2% of the patients presented to the emergency department within regular working hours (8:00 a.m.–6:00 p.m.) between Monday and Friday. Even though these data differ considerably from those in other studies [15], particularly because emergencies are simply unpredictable, the data collected show that a substantial proportion of the patients who present to the emergency department had the opportunity to present to a general practitioner or resident specialist for diagnosis and treatment, which could have a noticeable influence on the number of patients seen in the emergency department. In the present study, the majority of patients presented to our emergency department within the first 12 h (acute) after the trauma, but over 25% of the patients had experienced a trauma more than 24 h earlier, which would also have allowed them to present to a resident orthopedist/trauma surgeon. Regarding the frequent reason for presentation “distortion trauma” (14.8% of cases), the majority of patients (63.3%) presented during regular working hours. Again, a presentation could have taken place in the private practice sector, especially since nearly 90% of the distortion traumas did not reveal any acutely relevant trauma consequences. 

In the present study, the average age was approximately 36 years. A recently published study investigated why young people in particular sought emergency care in an emergency department relatively frequently, although for objectively “clinically unnecessary” reasons. These young adults stated reasons for going directly to the emergency department that included proximity to work or home and access to a faster appointment than they could obtain in the private practice sector, and also to minimize the negative impact on existing daily obligations, such as work or studies [16]. In addition, young adults quickly feel overwhelmed due to a new (and often first-time) unexpected health problem, which drives them to an urgent presentation at an emergency service [17]. A survey of patients presenting on their own to an emergency department showed that more than 90% of these patients considered themselves to be an emergency [12]. By contrast, in another survey study of patients presenting to the emergency department, more than half of the patients rated their reason for presentation as non-urgent, with patients presenting with musculoskeletal complaints in particular rating their treatment urgency as the lowest [3]. Similar results were shown in a publication by Unwin and colleagues, who identified musculoskeletal symptoms as the most common reason for presentation of non-urgent patients to the emergency department [18].

In particular, for the traumatological population, trauma room care for severely injured patients is a cornerstone of acute medical care. In line with the increasing patient load in the emergency department, an increase in patients presented through the traumatological trauma room was also observed [8]. However, the number of seriously injured patients has remained constant in recent years, so there is currently, on average, one seriously injured patient for every five trauma room admissions. Considering that, in addition to the structural requirements, trauma room care is also linked to a high demand for medical personnel, an increasing “over-triage” of trauma room patients leads to a further intensification of the already limited personnel resources and thus also to a further (over-)strain on the emergency departments. 

These factors can lead to crowding in the emergency department. On the other hand, crowding is forced by a lack of outflow of those patients who need to be hospitalized; however, at any given time, there is no bed capacity in the normal ward, and they must therefore remain in the emergency department for the time being [19]. In addition to economic effects, crowding also has direct consequences on patient care. Crowding leads to a longer hospital stay for patients [20]. In the 1990s, Krochmal et al. showed that patients who remained for a prolonged period in the emergency department after admission had an overall longer length of hospital stay than those who could be transferred immediately to a normal ward, which was significantly associated with higher costs to the health care system [21]. Crowding also leads to an increase in the incidence of medical errors [22]. For example, a prolonged stay in the emergency department in patients with a non-ST-segment elevation myocardial infarction was associated with lower adherence to guideline-recommended therapy and a higher risk of recurrent myocardial infarction [23]. Overall, crowding is associated with significantly increased patient mortality [20,24]. Currently, there are no data on this issue in a traumatological patient population.

The present studies illustrate that in the future, significantly more awareness needs to be raised regarding the availability of alternative outpatient emergency institutions in the general population in order to alleviate the burden on emergency departments. This will also require expansion of existing institutions, such as telephone contact centers and outpatient services and facilities for less urgent cases in or near hospitals with emergency departments. In particular, in view of the high percentage of musculoskeletal complaints, this may reduce the burden on emergency departments. Additionally, access to more “work-friendly” appointments with resident specialists as well as an overall faster appointment process in the outpatient sector needs to be provided. In order to avoid crowding in the emergency departments, the hospital infrastructure, such as bed management and discharge management in normal wards, needs to be further improved to ensure adequate outflow of patients in the face of already limited space and constrained personnel capacity. In this context, the overriding goals must be not only to maintain good medical care but also to reduce the individual psychological and physical stress on the staff working in the emergency department in order to continue to have motivated and efficient colleagues at the spearhead of acute care.

### Limitations

In this retrospective study, data from only one hospital in a metropolitan area were analyzed. The care of traumatological patients in smaller hospitals or even in smaller cities may differ from the data presented here. Furthermore, the available data were not analyzed over several years, but only for one complete year. Vacation periods and/or public holidays were not explicitly excluded. However, given the number of patients studied, the available data should provide a reliable representation of the situation in a traumatological emergency department of a maximum care university hospital. A strength of the present work is the individual evaluation of each patient. In this study, epidemiological data were analyzed in detail and not, as is often the case, by querying ICD (International Statistical Classification of Diseases and Related Health Problems) codes, which are of limited use for answering epidemiological questions.

## 5. Conclusions

In the context of the present study, we were able to demonstrate that a considerable portion of the traumatological patients presenting to the emergency department had met with an accident more than a day earlier. Common reasons for presentation, such as distortion trauma, turned out to be minor injuries in most cases. In order to conserve the precious resources of an emergency room, patients must be given better general awareness of the existing alternative outpatient care institutions. At the same time, the medical and nursing staff working there must be well-educated and trained in trauma surgery in order to recognize typical injury patterns and eventually treat them professionally. This also requires better networking and connections to practices with (native) radiological imaging, which is still lacking in non-hospital-based treatment. The available data show that, due to the high number of traumatological patients, the presence of trauma surgeons in the emergency department will remain essential for quality patient care in the future.

## Figures and Tables

**Table 1:** Time of trauma.

Time of Trauma	Number of Cases (n = 8582)
<12 h	5429 (63.3%)
12–24 h	833 (9.7%)
24–48 h	605 (7.1%)
48–72 h	486 (5.7%)
>72 h	1229 (14.2%)

The time of trauma is shown in relation to presentation to the emergency department (ED; n = 48,582). The majority (63.3%, n = 5429) of patients presented to the ED within the first 12 h after trauma (<12 h) or were presented by the ambulance service; 9.7% (n = 833) presented at 12 to 24 h post trauma, another 7.1% (n = 605) at more than 24 h, 5.7% (n = 486) at more than 48 h, and 14.2% (n = 1229) presented to the ED at more than 72 h after the trauma (>72 h) occurred.

**Table 2:** Presentation day.

Day of Presentation	Number of Cases (n = 8582)
Monday	1155 (13.5%)
Tuesday	1117 (13.0%)
Wednesday	1173 (13.7%)
Thursday	1032 (12.0%)
Friday	1211 (14.1%)
Saturday	1529 (17.8%)
Sunday	1365 (15.9%)

Shown are the frequencies of presentations by day of the week (n = 8582). Most emergency department presentations occurred on Saturdays (17.8%, n = 1529), followed by Sundays (15.9%, n = 1365), Fridays (14.1%, n = 1211), Wednesdays (13.7%, n = 1173), Mondays (13.5%, n = 1155), Tuesdays (13.0%, n = 1117), and Thursdays (12.0%, n = 1032).

**Table 3:** Reasons for presentation.

Reason for Presentation	Number of Cases (n = 8582)
Fall outdoors	1495 (17.4%)
Impact/bruise	1397 (16.2%)
Distortion	1270 (14.8%)
Fall indoors	854 (10.0%)
Cutting damage	714 (8.3%)
Pain without trauma	658 (7.7%)
Infection	525 (6.1%)
Bicycle fall	469 (5.5%)
Traffic accident	434 (5.1%)
Violence	302 (3.5%)
Other	464 (5.4%)

Shown are the various reasons that led to patient presentation to the emergency department (n = 8582). The most common reason for presentation was falls outside, followed by impact/bruise trauma and distortion trauma. “Other” includes, for example, lifting trauma, other wounds, and burns/scalds.

**Table 4:** Discharge diagnoses.

Diagnosis	Number of Cases (n = 8582)
Bruise	2366 (27.6%)
Fracture	1645 (19.2%)
Wound	1421 (16.6%)
Distortion	1194 (13.9%)
Radiculopathy	311 (3.6%)
Traumatic brain injury	273 (3.2%)
Tendinopathy	217 (2.5%)
Other	1155 (13.4%)

Shown are the frequencies of discharge diagnoses. The most common diagnosis recorded in the emergency department was bruise, followed by fractures, wounds, and distortions. Other diagnoses include, for example, polytrauma, luxation, burns/scalds, and erysipelas/phlegmons/abscesses.

## Data Availability

All data are contained within the article.
